# Empowering Medical Students: Harnessing Artificial Intelligence for Precision Point-of-Care Echocardiography Assessment of Left Ventricular Ejection Fraction

**DOI:** 10.1155/2023/5225872

**Published:** 2023-11-29

**Authors:** Ziv Dadon, Amir Orlev, Adi Butnaru, David Rosenmann, Michael Glikson, Shmuel Gottlieb, Evan Avraham Alpert

**Affiliations:** ^1^Jesselson Integrated Heart Center, Shaare Zedek Medical Center, Jerusalem, Israel; ^2^Faculty of Medicine, Hebrew University of Jerusalem, Jerusalem, Israel; ^3^Sackler Faculty of Medicine, Tel Aviv University, Tel Aviv, Israel; ^4^Department of Emergency Medicine, Shaare Zedek Medical Center, Jerusalem, Israel

## Abstract

**Introduction:**

Point-of-care ultrasound (POCUS) use is now universal among nonexperts. Artificial intelligence (AI) is currently employed by nonexperts in various imaging modalities to assist in diagnosis and decision making.

**Aim:**

To evaluate the diagnostic accuracy of POCUS, operated by medical students with the assistance of an AI-based tool for assessing the left ventricular ejection fraction (LVEF) of patients admitted to a cardiology department.

**Methods:**

Eight students underwent a 6-hour didactic and hands-on training session. Participants used a hand-held ultrasound device (HUD) equipped with an AI-based tool for the automatic evaluation of LVEF. The clips were assessed for LVEF by three methods: visually by the students, by students + the AI-based tool, and by the cardiologists. All LVEF measurements were compared to formal echocardiography completed within 24 hours and were evaluated for LVEF using the Simpson method and eyeballing assessment by expert echocardiographers.

**Results:**

The study included 88 patients (aged 58.3 ± 16.3 years). The AI-based tool measurement was unsuccessful in 6 cases. Comparing LVEF reported by students' visual evaluation and students + AI vs. cardiologists revealed a correlation of 0.51 and 0.83, respectively. Comparing these three evaluation methods with the echocardiographers revealed a moderate/substantial agreement for the students + AI and cardiologists but only a fair agreement for the students' visual evaluation.

**Conclusion:**

Medical students' utilization of an AI-based tool with a HUD for LVEF assessment achieved a level of accuracy similar to that of cardiologists. Furthermore, the use of AI by the students achieved moderate to substantial inter-rater reliability with expert echocardiographers' evaluation.

## 1. Introduction

Point-of-care ultrasound (POCUS) is frequently utilized by physicians in many medical specialties as well as among medical students [1]. With the recent development of low-cost portable devices and increasing number of applications, it is expected that POCUS use will expand in the coming years [2, 3]. Furthermore, in fields such as emergency medicine or internal medicine, the expected results are often general or binary (i.e. pericardial fluid present or absent; left ventricular ejection fraction (LVEF) normal or grossly abnormal) rather than a detailed result given by a cardiologist [4, 5]. Also, different POCUS guidelines have been proposed with basic requirements including a qualitative assessment of left ventricular (LV) systolic function, leaving the exact calculation of LVEF to expert echocardiographers only [6]. The specific quantification of cardiac LVEF is one of the most significant and frequent applications of echocardiography [7]. Nonetheless, the methods used to make these specific calculations are operator-dependent [8].

Artificial intelligence (AI) and computational technologies are increasingly utilized across various imaging modalities, including cardiac imaging, by nonexpert operators. They assist in decision making and enhancing diagnostic capabilities [9–12]. In the clinical practice of echocardiography, AI is mainly used in automated tools implemented in high-end devices but less incorporated in systems used for point-of-care testing [13]. This is due to the low frame rate and image quality that limit the use of speckle-tracking algorithms on these devices. Incorporating AI into real-time focused echocardiography operated by noncardiologists to accurately assess various cardiac functions may significantly improve accurate image interpretation, reduce variability among nonexperts, and lead to better diagnostic decisions.

The objective of this study was to assess the diagnostic accuracy of an AI-based assessment tool on a hand-held ultrasound device (HUD) operated by medical students as compared with cardiologists' visual evaluation in assessing the LVEF of patients hospitalized in a cardiology department of a tertiary care teaching hospital.

## 2. Methods

### 2.1. Study Design

This was a prospective study of real-time focused echocardiography operated by medical students using an AI-based technology for LVEF evaluation compared to cardiologists and expert echocardiographers. The study was approved by the hospital's Institutional Review Board (IRB number: 0325-18-SZMC).

**Figure 1:**
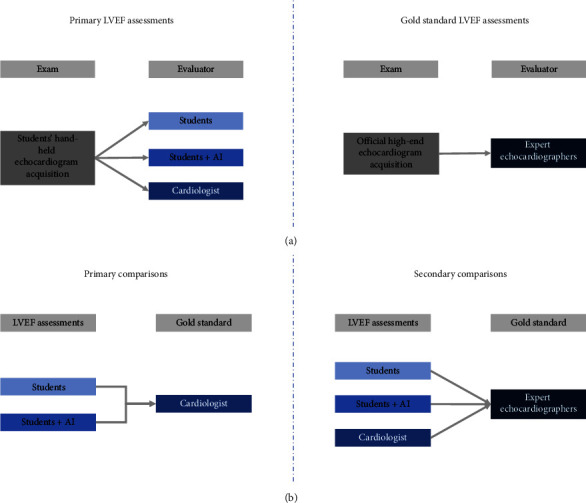
Flowchart of LVEF assessments and the primary and secondary comparisons. (a) The LVEF assessment methods using the students' HUD-acquired clips upon recruitment to the study and the echocardiographer assessment using the formal high-end echocardiography clips completed within 24 hours from recruitment to the study. (b) The study's primary comparison included LVEF assessment on students' HUD-acquired clips: students vs. cardiologists and students + AI vs. cardiologists. The secondary comparisons included the three assessment methods of students' HUD-acquired echocardiography clips (students' visual evaluation, AI + students, and cardiologists' visual evaluation) with the fellowship-trained expert echocardiographer's assessment of the formal high-end echocardiography. AI, artificial intelligence; HUD, hand-held ultrasound device; LVEF, left ventricular ejection fraction.

The clips acquired using the HUD were assessed for LVEF by three methods: visually by the students, students + the AI-based tool, and visually by cardiologists (Figure 1(a)). A formal echocardiography was completed within 24 hours including LVEF eyeballing and Simpson's method evaluation by expert echocardiographers (Figure 1(b)).

### 2.2. Study Comparisons

The study's primary comparison was designed to show that the AI-based tool used by nonexperts for LVEF evaluation is accurate compared with cardiologists' assessment. A correlation goal of 80% between the AI-based tool and the cardiologists was defined as suitable for the study.

Secondary comparisons included all three LVEF measurements (students, students + AI-based tool, and cardiologists) as compared with a parallel mean assessment of the high-end echocardiography completed within 24 hours by the expert echocardiographers.

### 2.3. Patient Selection

Study participants were nonselected patients admitted to the cardiology department within their first 48 hours of hospitalization. As part of the cardiology department routine, all admitted patients underwent an official echocardiography within the first 24 hours of hospitalization.

### 2.4. Study Setting

**Figure 2:**
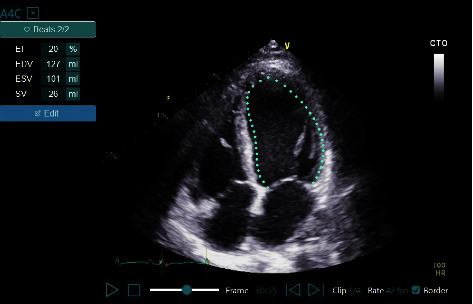
LVivo EF: AI-based tool for automated LVEF assessment from apical 4-chamber view echocardiographic clips. AI, artificial intelligence; LVEF, left ventricular ejection fraction.

The study was conducted at a single tertiary care medical center from March 2019 through March 2020 and included 4^th^ to 6^th^ year medical students that routinely worked in the cardiology department as physician assistants. The students were trained to use a HUD (Vscan Extend with Dual Probe; General Electric) equipped with LVivo EF (DiA Imaging Analysis Ltd), an AI-based program, that provides automated calculation of LVEF from the apical 4 chamber (A4ch) view (Figure 2). The students were assigned to read preliminary relevant information after which they underwent a quiz to assess their knowledge. They then underwent a 6-hour course that included frontal lectures and hands-on practice. The frontal lectures discussed background information, practical information, and heterogeneous echocardiographic video clips encompassing the full clinical range of LVEF calculation. During the hands-on practice, each of the participants had to complete at least four supervised scans assessed for both proper acquisition and LVEF evaluation. Prior to the clinical study, a preliminary practical examination of the devices and the AI-based application was performed by the principal investigators for troubleshooting and to rule out any practical problems. Following the training, a pilot phase was conducted where the operators' skills were evaluated. A total of nine students were trained in the course, and one did not complete the required training, leaving eight students who participated in the study.

### 2.5. Study Protocol

Written consent was obtained from all patients who participated in the study. Those who refused to participate or whose AI-based measurement was unsuccessful were excluded. Data included age, sex, body mass index (BMI), relevant chronic comorbidities, and admission presentation. Technical aspects were also recorded including the patient's ability to turn on their left side, their proficiency in maintaining effective communication (i.e. adhering to instructions and cooperating with the examination), study difficulty, and quality.

The study flowchart is shown in Figure 1(a). The medical students performed the POCUS examination using the HUD and acquired the echocardiography clips obtained from the A4ch view. The study acquisition was evaluated by the students on a scale of 1–3 for difficulty (easy, intermediate, or difficult) and on a scale of 1–4 for image quality (excellent—optimal visualization; high—proper visualization of >50% of the segments; moderate—<50%; and poor—inappropriate visualization). The view was focused and optimized on the LV, avoiding foreshortening. The interventricular septum was aligned parallel to the plane, and at least a 2-beat heart cycle was recorded. Depth was adjusted so that the LV occupied two-thirds of the view. The students were then asked to visually evaluate the exact LVEF. Next, the acquired clips were visually evaluated by a cardiologist (AO and ZD), who were blinded to the previous results, for a second LVEF measurement and image quality according to the abovementioned scale. Finally, the LVEF was assessed on the recorded echocardiographic clips using the AI-based application (after both the student and cardiologist have committed to specific LVEF values). In case of a failure of the automated algorithm to calculate the LVEF, if the entire border tracings were incorrect or if the clip was significantly foreshortened, the image acquisition was repeated (up to five subsequent attempts).

The patients underwent an official echocardiogram using a high-end device within 24 hours of being recruited into the study. These clips were acquired by a certified echocardiographic technician (equivalent to a Registered Diagnostic Cardiac Sonographer in the United States). Each formal echocardiogram was evaluated for LVEF using both visual evaluation and Simpson's method by two fellowship-trained expert echocardiographers (AB and DR), blinded to the patient's details and previous study assessments.

### 2.6. Data Management

Following consent, the study patients were given a separate anonymous identifying number for the study documentation. The Primary Investigator (PI) kept an Excel file with the case identifying number, the date of the study, and patient identifiers (*PI file*). The HUD-based LVEF results were inserted into a second file using the patient's identifying number (*Hand-held file*). Official echocardiography results were documented on a third file (*Official file*). The HUD results were later matched to official results using the identifying number.

### 2.7. Sample Size Calculation

Sample size calculations were designed to meet the study comparison and were performed using G∗Power software (version 3.1.9.4, Heinrich Heine University Düsseldorf, Germany). We planned a paired study with a 1 : 1 ratio. While previous data regarding LVivo EF usage showed a high correlation with the gold standard [14], in order to maximize data yield, we assumed a low correlation of 0.4 between the AI-based LVEF calculation and the cardiologist's assessment. Based on these assumptions, we calculated that data accrued from 67 participants would suffice to reject the null hypothesis with a probability (power) of 0.9. Type I error was calculated as 0.05 and was two-tailed.

### 2.8. Statistical Analyses

Descriptive statistics were used to analyze baseline and clinical characteristics as well as echocardiography results and comparisons, using chi-square or Fisher's exact tests for categorical variables, and the *t*-test or Mann–Whitney *U* test for continuous variables, where appropriate test selection was based on data distribution and normalcy.

For continuous LVEF comparisons, the paired *T*-test or signed-rank test for two means (paired observations) were applied to test the statistical significance of the differences between the results obtained from each method.

The students' visual evaluation and the students + AI-based tool LVEF continuous evaluations were compared to the cardiologists' assessment for linear correlation using the Pearson correlation coefficient (*r* values <0.3, 0.3 to 0.5, 0.5 to 0.7, and ≥0.7 were considered to represent poor, poor to fair, fair to good, and excellent correlation, respectively). LVEF assessment agreement and bias were calculated using the Bland–Altman analysis including mean difference and 95% limits of agreement (according to 2 standard deviations).

For categorical variables, the inter-rater reliability using the Kappa coefficient was then calculated using cutoffs of 50% and 40% for the LVEF between the echocardiographer's high-end device assessment and the three HUD-based LVEF evaluations, including visually by the students, students + AI tool, and visually by the cardiologists. Kappa values 0, 0 to 0.2, 0.21 to 0.40, 0.41 to 0.60, 0.61 to 0.80, and ≥0.81 were considered to represent no agreement, slight, fair, moderate, substantial, and almost perfect agreement, respectively.

All tests were two-tailed, and a *p* value of 5% or less was considered statistically significant.

Statistical analyses were performed using SPSS Statistics for Windows version 26 (SPSS Inc., Chicago, IL).

## 3. Results

A total of 88 patients were recruited for the study. The AI-based automatic measurement was not successful for six patients, leaving 82 patients for inclusion in the analysis. The mean age of the included cohort was 58.5 ± 16.8 years with 72 (87.8%) male participants.

### 3.1. Baseline Demographic and Clinical Characteristics: Successful vs. Unsuccessful AI Measurement (Table 1)

Patients with unsuccessful AI measurement, as compared to those with successful measurements, had studies of lower quality (100% with moderate/poor quality vs. 52.4%, *p*  <  0.001), greater difficulty in study performing (83.3% difficult studies vs. 32.9%, *p* = 0.040), longer study durations (14.7 ± 3.4 vs. 6.3 ± 3.1 minutes, *p*  <  0.001), and lower mean LVEF (38.9 18.5 vs. 52.1 ± 11.3%, *p* = 0.038).

### 3.2. Correlation of the LVEF Assessment Methods: Students vs. Cardiologists and Students + AI vs. Cardiologists

**Figure 3:**
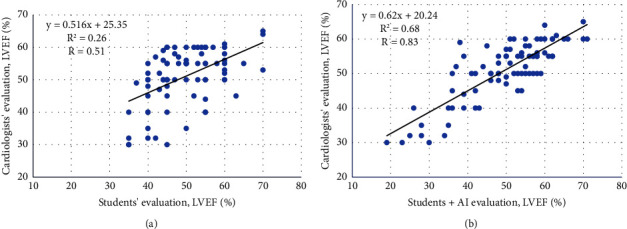
Correlation of LVEF assessment on students' HUD-acquired clips: students vs. cardiologists and students + AI vs. cardiologists. (a) Correlation of LVEF assessment: students' visual evaluation vs. cardiologists (Pearson correlation of 0.51, *p*  <  0.001). (b) Correlation of LVEF assessment: students + AI vs. cardiologists (Pearson correlation of 0.83, *p*  <  0.001). AI, artificial intelligence; HUD, hand-held ultrasound device; LVEF, left ventricular ejection fraction.

A fair to good correlation was demonstrated between the students' and the cardiologists' visual evaluation for LVEF assessment of the students' acquired clips, with a Pearson correlation coefficient of 0.51 (*p*  <  0.001; Figure 3(a)). An excellent correlation was demonstrated between the students + AI measurement and the cardiologists' visual evaluation for LVEF assessment, with a Pearson correlation coefficient of 0.83 (*p*  <  0.001; Figure 3(b)).

### 3.3. Assessment Agreement of the LVEF Assessment Methods: Students vs. Cardiologists and Students + AI vs. Cardiologists

**Figure 4:**
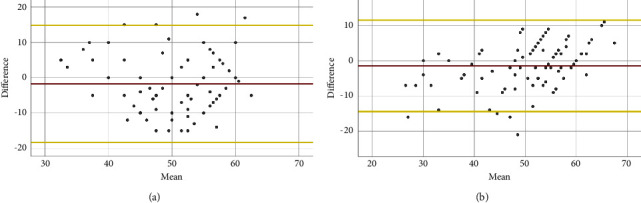
The agreement using the Bland–Altman analysis of LVEF assessment on students' HUD-acquired clips: students vs. cardiologists and students + AI vs. cardiologists. (a) LVEF assessment agreement between the students' and cardiologists' visual evaluation revealed a mean bias of −1.77 (red line) with limits of agreement ranging from −18.37 to 14.83 (yellow lines), *p* = 0.062. (b) LVEF assessment agreement between the students + AI and cardiologists' visual evaluation revealed a mean bias of −1.44 (red line) with limits of agreement ranging from −14.40 to 11.52 (yellow lines), *p* = 0.052. AI, artificial intelligence; HUD, hand-held ultrasound device; LVEF, left ventricular ejection fraction.

LVEF assessment agreement between the students' and the cardiologists' visual assessment of the HUD-acquired clips using the Bland–Altman analysis revealed a mean bias of −1.77 (*p* = 0.062), with limits of agreement ranging from −18.4 to 14.8 (Figure 4(a)). LVEF assessment agreement between the AI measurement and the cardiologists' visual assessment of the students' acquired echocardiography clips revealed a mean bias of −1.44 (*p* = 0.052), with limits of agreement ranging from −14.4 to 11.5 (Figure4(b)).

### 3.4. Inter-Rater Reliability of LVEF Assessment: The Three Assessment Methods of HUD-Acquired Clips vs. the Expert Echocardiographers' Assessment of High-End Device Clips

**Figure 5:**
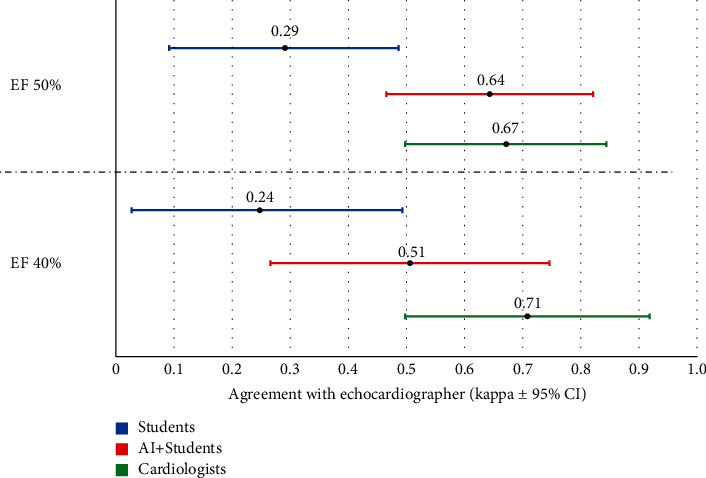
Categorical inter-rater reliability of LVEF assessment using Kappa coefficient comparing the 3 assessment methods of students' HUD-acquired echocardiography clips (students' visual evaluation, AI + students, and cardiologists' visual evaluation) with the fellowship-trained expert echocardiographers' assessment of the formal high-end echocardiography using 2 cutoff values of LVEF for each set of analyses (LVEF of 40 and 50%). AI, artificial intelligence; CI, confidence interval; HUD, hand-held ultrasound device; LVEF, left ventricular ejection fraction.

As shown in Figure 5, the categorical agreement of LVEF assessment comparing the three assessment methods of students' acquired clips (students, students + AI, and cardiologists) with the expert echocardiographers' assessment of the formal echocardiogram using LVEF 50% as cutoff revealed a substantial agreement for the AI measurement and the cardiologists (Kappa of 0.64, standard error of 0.09, *p*  <  0.001 and Kappa of 0.67, standard error of 0.09, *p*  <  0.001, respectively) but only a fair agreement for the students' visual evaluation (Kappa of 0.29, standard error of 0.10, *p* = 0.007). A similar analysis using LVEF 40% as the cutoff revealed a moderate agreement for the AI measurement (Kappa of 0.51, standard error of 0.12, *p*  <  0.001) and a substantial agreement for the cardiologists (Kappa of 0.71, standard error of 0.10, *p*  <  0.001) but a fair agreement for the students' visual evaluation (Kappa of 0.24, standard error of 0.13, *p* = 0.027).

## 4. Discussion

This study showed that the use of an AI-based tool on a HUD operated by medical students for LVEF assessment of patients admitted to the cardiology department has a high correlation with cardiologist visual assessment. Moreover, when compared with fellowship-trained expert echocardiographers using a high-end device, the AI-based LVEF measurement of the students' HUD-acquired clips can reach an agreement significantly higher than student visual evaluation and almost as good as that of the cardiologists.

The increasing use of POCUS by clinicians across specialties has been accompanied by a parallel introduction of ultrasound to medical students [15]. However, according to one critical systematic review, ultrasound was not shown to improve medical students' understanding of anatomy and only some studies show that it improves diagnostic abilities while there are no clear benefits in terms of patient outcomes [16]. Many of the tools suggested to enhance ultrasound skills involve either passive learning or are not conducted in clinical settings [17, 18].

As POCUS has gained popularity across many medical disciplines, the use of HUD has expanded due to its advantages, including small size, portability, cost, and its ability to provide a real-time and instantaneous assessment [19]. These characteristics were proven useful in settings that can lead to a direct impact on immediate patient diagnosis and management and led to HUD utilization for bedside evaluations, including during the COVID-19 pandemic [20–22]. Though HUD use may involve several limitations, including screen size, imaging quality, and equivocal observations, its utilization was found to be reliable and accurate in different POCUS settings if properly performed [23, 24]. Similarly, this study demonstrated a moderate/substantial agreement between a HUD with an AI-based tool operated by medical students and high-end devices operated by skilled sonography technicians and evaluated by expert echocardiographers.

Short-term accurate assessment of LVEF by medical students following a dedicated training session has been previously shown for both prerecorded and real-time acquired clips [25, 26]. In contrast to these studies, our research took place in a real-time setting on patients admitted to the cardiology department and included both independent clip acquisition and LVEF evaluation by medical students. Also, the long-term effect of training for echocardiography diagnosis among novice users who are not routinely exposed to echocardiography practice is less studied and is not always maintained [27]. Aside from the obvious loss of training that increases with time and lack of reinforcement, this observation may also stem from a limited training effect secondary to novice users' adoption of a less structured approach to image reading ending with a less efficient analysis. This trend may explain the finding in the present study that, unlike previous publications [25, 26], the diagnostic capability of LVEF by the students was suboptimal when based only on visual evaluation. These findings suggest that not only an objective assessment of real-time acquisition and interpretation of echocardiographic data should be incorporated into the assessment of proficiency of novice users in echocardiography, but training programs should also provide a long-term environment for skill maintenance.

The AI-based tool used in the present study (*LVivo EF*) has been recently validated using a traditional formal echocardiography device for LVEF automated quantification as compared with cardiac magnetic resonance imaging [28]. Also, a previous study that tested this AI tool use with hand-held echocardiography clips acquired by a 5th year cardiology resident found an excellent correlation of 0.92 for the entire studied cohort as compared with formal echocardiography [14]. Similarly, we found a correlation of 0.82 with the cardiologist evaluation as well as a high agreement when compared with fellowship-trained expert echocardiographers. While Filipiak-Strzecka et al. tested the tool on a single highly trained cardiology resident (after six months of training in the echocardiography lab), the echocardiography acquisition in the present study was conducted by eight medical students who underwent only a 6-hour didactic course, and as such it reflects real-life novice use.

We have shown that all of the cases with unsuccessful AI measurement had a poor/moderate quality, were difficult to perform, and had a lower LVEF as compared with the successful cases. As the phased array transducer on this particular HUD is narrower than in conventional devices, if the LV was severely dilated, it may be challenging to include all borders in view throughout the entire cardiac cycle, resulting in an unsuccessful AI measurement. Similar to the findings of this study, Filipiak-Strzecka et al. found that most of the unsuccessful AI tool calculations were conducted on poor-quality clips (26/36). Notably, they showed an unsuccessful AI measurement in 36 patients (27% of those attempted), whereas in our study, the rate was only 7% (6/88). Samtani et al. showed a 2% (6/242) unsuccessful rate of AI-based measurement using a standard echocardiography device [28]. The varying rate of unsuccessful attempts may result from a higher number of acquisition attempts (five vs. three) or from differing patients and image characteristics including a lower volume of poor image quality (7 vs. 23%) and a potentially higher volume of normal functioning hearts (the proportion in their study was not published).

### 4.1. Limitations

The study extended over one year, resulting in a gradual loss of training since the didactic course. This factor may account for the relatively lower diagnostic accuracy observed among the medical students. Nonetheless, they retained their acquisition capabilities as proven by the comparisons to the high-end device evaluations. Moreover, the study reflects real-life clinical practice, as novice users are utilizing their skills for months and years after their initial training. A significant limitation is that the cardiologist visual assessment used as the reference was done on the students' acquired clips and could have been foreshortened. This design was chosen to minimize the potential biases for LVEF mismatch, including different acquisition by experienced personnel. Another limitation is the relatively small sample size from a single medical center. Even though this study compared different echocardiographic methods for the assessment of LVEF, i.e., visually estimated evaluation vs. tracing of the ventricular borders and exact maximum and minimum surface measurement (via the AI-based tool and Simpson's method), it has been shown that the two are closely correlated when properly conducted [29]. Moreover, the scales used for acquisition difficulty and image quality were not based on official guidelines and were created for study purposes. Also, the LVEF evaluation was assessed by the students using the A4ch view exclusively. Nonetheless, the LVEF evaluation was accurate with a moderate/substantial agreement achieved when compared with the high-end device clips assessed from all views.

## 5. Conclusions

Medical students can improve their LVEF assessment proficiency using a HUD to match that of cardiologists through the utilization of an AI-based tool. In addition, the use of AI for LVEF assessment enabled novice users to achieve moderate to substantial inter-rater reliability as compared with expert echocardiographers. This study offers a rationale for considering the use of this AI-based tool as an effective decision-making support tool for POCUS LVEF evaluation by nonexperts. Further studies should be conducted among different types of noncardiologist clinicians such as internists, emergency physicians, and physician assistants to assess the generalizability of these findings. In addition, prospective studies should be conducted to investigate whether AI-based tools can impact patient outcome.

## Figures and Tables

**Table 1:** Baseline demographics and clinical characteristics of those with successful AI vs. unsuccessful AI measurements.

Variable	Successful AI measurement *n* = 82	Unsuccessful AI measurement *n* = 6	*p* value
*Baseline demographic and clinical characteristics*			
Age (years), mean ± SD	58.5 ± 16.8	54.7 ± 7.1	0.578
Male, *n* (%)	72 (87.8)	4 (66.7)	0.188
BMI, mean ± SD	28.2 ± 4.5	29.8 ± 3.6	0.397
Smoking, *n* (%)	46 (56.1)	4 (66.7)	0.214
Ability to turn left, *n* (%)	74 (90.2)	6 (100)	0.886
Effective communication, *n* (%)	78 (95.1)	6 (100)	0.703
Active dyspnea, *n* (%)	77 (93.9)	6 (100)	1.000
Lung disease, *n* (%)	79 (96.3)	5 (83.3)	0.140

*Exam quality* ^+^			**<0.001**
Excellent, *n* (%)	15 (18.3)	0 (0.0)	
High, *n* (%)	24 (29.3)	0 (0.0)	
Moderate, *n* (%)	41 (50.0)	2 (33.3)	
Poor, *n* (%)	2 (2.4)	4 (66.7)	

*Exam difficulty* ^*∗*^			**0.040**
Easy, *n* (%)	29 (35.4)	0 (0.0)	
Intermediate, *n* (%)	26 (31.7)	1 (16.7)	
Difficult, *n* (%)	27 (32.9)	5 (83.3)	

*Admission diagnosis*			0.432
Heart failure, *n* (%)	9 (11.0)	2 (33.3)	
Acute coronary syndrome, *n* (%)	48 (58.5)	3 (50.0)	
Arrhythmia, *n* (%)	11 (13.4)	0 (0.0)	
Perimyocarditis, *n* (%)	8 (9.8)	1 (16.7)	
Other, *n* (%)	6 (7.3)	0 (0.0)	

*Exam setting*			
HR (bpm), mean ± SD	73.9 ± 13.3	85.0 ± 18.5	0.056
Sinus rhythm, *n* (%)	73 (89.0)	6 (100)	0.513
Length of study (minutes), mean ± SD	6.3 ± 3.1	14.7 ± 3.4	**<0.001**
Normal LVEF, *n* (%)^++^	42 (51.2)	2 (33.3)	0.676
LVEF (%), mean ± SD^++^	52.1 ± 11.3	38.9 ± 18.5	**0.038**

^**+**^As per cardiologist evaluation. ^*∗*^As per medical student evaluation. ^++^As per expert echocardiographer assessment of the formal echocardiography. AI, artificial intelligence; BMI, body mass index; bpm, beats per minute; HR, heart rate; LVEF, left ventricular ejection fraction; *n*, number; SD, standard deviation. Bold values indicate the *p* value is statistically significant (less than 0.05).

## Data Availability

The anonymized data used to support the findings of this study may be released upon application to the corresponding author.

## Abbreviations

A4ch: Apical 4 chamber
AI: Artificial intelligence
BMI: Body mass index
HUD: Hand-held ultrasound device
IRB: Institutional Review Board
LV: Left ventricular
LVEF: Left ventricular ejection fraction
POCUS: Point-of-care ultrasound.
